# The delivery of radical radiotherapy to the bladder and pelvis in node-positive (N1) bladder cancer: a five patient case series

**DOI:** 10.1259/bjrcr.20160102

**Published:** 2016-12-23

**Authors:** David Gareth Fackrell, Daniel Ford, Shan Chetiyawardana, Samantha Austin, Nicholas James

**Affiliations:** The Cancer Centre, University Hospital, Birmingham, Queen Elizabeth, UK

## Abstract

In the UK over 10,000 new cases of bladder cancer were diagnosed in 2012, making it the seventh most common cancer in the UK. For those with advanced disease at presentation, prognosis is poor. Disease presenting with one pathological node (N1) is considered to be Stage 4 and is therefore considered the same as disease with widespread metastases. Pelvic lymph node dissection is considered standard when performing radical cystectomy; however, owing to potential toxicity, pelvic radiotherapy is not routine even when attempting radical treatment. We present five cases where radical treatment has been delivered to patients with node-positive bladder cancer. Treatment volumes included the whole bladder and bilateral pelvic nodes, and where it was felt appropriate, chemotherapy was delivered concurrently. Data has been collected by reviewing hospital notes including radiotherapy, volumes and dose distributions. Treatment was tolerated well with only minimal gastrointestinal and urinary symptoms reported. Three of the five patients had thrombocytopenia. This complication may be explained by the larger volume of radiation exposure. Local control appears to be good with all the patients having no pelvic relapse at the time of reporting. Two patients have relapsed with distant metastatic disease. No long-term side effects of therapy have been reported. Intensity-modulated radiotherapy techniques allow larger volumes to be treated owing to improved conformality and the resulting reduced toxicity. Treatment may be appropriate with both radical and adjuvant doses of radiation. More work is necessary to assess which patients would benefit and are most suitable for such treatment.

In the UK over 10,000 new cases of bladder cancer were diagnosed in 2012, making it the seventh most common cancer in the UK accounting for 3% of all new cases in the UK.^1^ The majority of cases are diagnosed at an early, superficial stage but for those with more advanced disease at presentation the prognosis is far worse. Disease presenting with one pathological node (N1) is considered by the American Cancer Society to be Stage 4 and is, therefore, classed the same as disease with widespread metastases. Despite the use of neoadjuvant and adjuvant chemotherapy to improve outcomes post-operatively, 5-year survival for Stage 4 disease is reported to be far worse than that for Stage 3 (15% versus 46%).^2^

Despite the significantly increased mortality and potential increased morbidity, pelvic lymph node dissection is considered standard when performing radical cystectomy. Patients with lymph node involvement are considered to have a poorer prognosis after radical cystectomy and pelvic lymph node dissection but survival rates do vary. However, improvements in surgical outcomes have been reported in several retrospective studies.^3–5^ Controversy exists regarding the optimal extent of the lymph node dissection that should accompany cystectomy. However, it is clear that at a minimum, a standard dissection should be performed.

The optimum strategy for the control of bladder cancer is undetermined, and radical radiotherapy, with or without concurrent chemotherapy, allows for bladder sparing and excellent local control rates. For example, James et al^6^reported local control rates of 67% at 2 years when concurrent chemotherapy was used with radical radiation doses. Such good control rates were observed without treating the pelvic nodes even to a prophylactic dose. Two dose levels were used in this study, 55  Gy in 20 fractions over a 4-week period or 64 Gy in 32 fractions over a 6.5-week period. Radiotherapy was planned and delivered using a field-based technique, for example, three to four coplanar fields covering the planned target volume (PTV) around the bladder alone to 95% of the prescribed dose. This treatment was well tolerated, with grade 3 or 4 Radiation Therapy Oncology Group (RTOG) adverse events occurring during follow-up in only 8.3% and 15.7% of patients in the chemoradiotherapy and radiotherapy groups respectively. At 1 year, grade 3 or 4 RTOG adverse events were reported in only 3.3% in the chemoradiotherapy group and 1.3% in the radiotherapy group.

Three-dimensional conformal techniques for pelvic radiotherapy are associated with morbidity that is dose related. This limits the dose of radiation that can be prescribed as bowel toxicity can be significant. Intensity-modulated radiotherapy (IMRT) techniques can substantially reduce the bowel and bladder volume irradiated during pelvic radiotherapy. Dose escalation to the pelvic region is therefore possible. IMRT is an advanced method of radiotherapy allowing linear accelerators to deliver precise radiation doses to specific areas and allows for the radiation dose to conform more accurately to the shape of the tumour. The intensity of the radiation is modulated within multiple beams, thereby allowing higher radiation doses to be focused on regions within the tumour; smaller prophylactic doses can be simultaneously delivered to high risk area and the dose to surrounding normal critical structures can be minimized. The technique is now considered as the gold standard for treating tumours of the head and neck region and is being used with increasing frequency in other areas such as the pelvis. For example, in the Prostate and pelvIc Versus prOstaTe ALone (PIVOTAL) trial prostate and pelvic nodal radiotherapy is compared to treatment to the prostate alone using high-dose IMRT in patients with locally advanced prostate cancer. The use of IMRT has allowed a larger volume to be treated without significant compromise on toxicity. The volume of bowel and colon irradiated to the 90% isodose level is reduced from 24% using conventional radiotherapy to 18% using conformal techniques but to only 5% using IMRT.^7^ By reducing the dose to the bowel, IMRT reduces treatment-related complications, allowing dose escalation to at-risk lymph node regions.

Nodal radiotherapy in the treatment of prostate cancer is becoming increasingly common. In the RTOG 94-13 trial, pelvic radiotherapy to a dose of 50.4 Gy in 1.8 Gy fractions improved progression-free survival in selected, high-risk patients.^8^ Phase I and II dose escalation studies of prostate plus pelvic lymph node radiotherapy in patients with high-risk prostate cancer showed that doses of up to 60 Gy to the pelvis were safe when IMRT was used. Patients treated with radiotherapy received 70 Gy in 35 fractions to the prostate and seminal vesicles and 50 (n = 25), 55 (n = 55) or 60 Gy (n = 135) to the pelvic lymph node region. Acute and late toxicity rates in the 50 and 55 Gy groups were low. In the 60 Gy group, acute (RTOG grade ≥2) bladder and bowel toxicity was a little higher (4.5% at week 18 of follow-up).^9^ These toxicity data have established the feasibility to allow the setting up of the PIVOTAL trial to investigate nodal radiotherapy in prostate cancer but also support the potential use of similar techniques and doses for other cancer sites.

There is potential benefit by treating the pelvic nodes to a prophylactic, or in some cases, radical dose of radiation. With IMRT, pelvic radiation is now more tolerable and therefore, we present five cases where radical treatment has been delivered to patients with node-positive bladder cancer. Treatment volumes included the whole bladder and bilateral pelvic nodes, and where it was felt appropriate, chemotherapy was delivered concurrently. Data has been collected by reviewing hospital notes including radiotherapy, volumes and dose distributions.

## Patient case series

### Case 1

A 79-year-old male presented with locally advanced transitional cell carcinoma (TCC) of the bladder. This was staged as T2 N1 M0 radiologically. Transurethral resection of the bladder tumour was performed and he was then treated with three cycles of gemcitabine and cisplatin chemotherapy. Post chemotherapy cystoscopy revealed normal appearances of the urothelium and bladder capacity of 400 ml. Post chemotherapy CT imaging showed almost total resolution of the pelvic lymphadenopathy.

The patient was treated with 64 Gy in 32 fractions over 6.5 weeks to his bladder together with 53 Gy in 32 fractions to his pelvic nodes at the same time. He received synchronous 5-flurouracil (5-FU) and mitomycin-C (MMC) in weeks 1 and 4 of treatment.

Treatment was tolerated well with only mild, occasional diarrhoea. Full blood counts were largely normal despite the large volume irradiated. Platelet count was reduced with a minimum value of 82 × 10^9^ l^–1^ in week 3 of treatment.

This patient has been followed up for 17 months and at that time remained disease and symptom free. A cystoscopy showed mild radiation changes throughout the bladder only.

### Case 2

A 77-year-old male presented with a background of metastatic prostate cancer. At the time of diagnosis with a TCC of the bladder his prostate cancer was well controlled with androgen deprivation therapy. He presented with haematuria, and cystoscopy identified muscle invasive disease. A single involved obturator node was identified on imaging that had developed alongside the bladder cancer while the prostate disease had been well controlled. He was therefore staged at T3 N1 M0.

The patient received three cycles of neoadjuvant gemcitabine and cisplatin chemotherapy following transurethral resection of the bladder tumour. Post treatment cystoscopy showed only scarring where the tumour had been excised. His CT imaging showed that the obturator node had regressed significantly.

The patient subsequently received concurrent chemoradiotherapy. 64 Gy was delivered in 32 fractions with concurrent 5-FU and MMC. The patient tolerated therapy well. No significant toxicities were reported. He did however, experience thrombocytopenia, with the lowest recorded result 92 × 10^9^ l^–1^.

Nineteen months after completion of his treatment, the patient remains well. No long-term consequences of his therapy have been reported and he has had no relapse of his bladder cancer as monitored by CT imaging and cystoscopy.

### Case 3

A 74-year-old male with T3 bladder TCC with left internal iliac and obturator lymph node positivity presented with hydronephrosis. This was treated with the insertion of a nephrostomy before neoadjuvant gemcitabine and cisplatin chemotherapy was started.

Following three cycles of chemotherapy a CT scan showed excellent response with complete resolution of the previously identified pelvic lymphadenopathy.

The patient then received radical radiotherapy, 64 Gy in 32 fractions to the bladder with 53 Gy in 32 fractions to pelvic lymph nodes. Treatment was delivered concurrently with 5-FU and MMC chemotherapy in week 1 only. Week 4 chemotherapy was cancelled owing to thrombocytopenia (platelets of 86 × 10^9^ l^−1^). The patient otherwise tolerated therapy well with no other side effects reported.

A follow-up scan 6 months after treatment showed no evidence of metastatic disease or local relapse. No permanent sequelae of his therapy are reported.

### Case 4

A 67-year-old female presented with a high-grade transitional cell carcinoma after presenting with cystitis. Initial staging was T2 N0 and the patient underwent three cycles of neoadjuvant gemcitabine and cisplatin chemotherapy before a restaging scan showed two enlarged iliac lymph nodes despite an otherwise good response to treatment. The multidisciplinary meeting at the treating hospital agreed that these lymph nodes were likely to be malignant and therefore the patient went on to have radiotherapy where the treatment volume included the pelvic nodes also. 64 Gy in 32 fractions was delivered to the bladder, the suspicious pelvic node received 52 Gy in 27 fractions and the bilateral nodal volume received 48 Gy in 27 fractions. MMC and 5-FU chemotherapy was given concurrently.

The patient tolerated the therapy extremely well. No detrimental fall in the full blood count was observed and all chemotherapy was delivered as planned. She complained of no acute radiation toxicity other than mild lethargy and diarrhoea.

Unfortunately, while her post treatment restaging scan showed that the pelvic lymphadenopathy had regressed, there was significant lymphadenopathy outside the radiotherapy volume in the retroperitoneum, mediastinum and both sides of the neck. A biopsy confirmed this to be metastatic TCC.

The patient went on to receive vinflunine chemotherapy as the standard treatment arm in a clinical trial. Despite an initial good response, chemotherapy had to be discontinued owing to neutropenia. The patient developed metastaic liver lesions and was started on weekly paclitaxel, which continues for 9 months after completing radical treatment.

### Case 5

A 70-year-old male of previous excellent health presented with haematuria and was found to have T2 N1 TCC of the bladder. He underwent four cycles of neoadjuvant chemotherapy with gemcitabine and cisplatin without complication and was shown to have had a good response. He was felt to be inoperable owing to the node-positive state and was therefore considered for radiotherapy.

The patient received 64 Gy in 32 fractions to the bladder and positive nodes. A prophylactic dose of 53 Gy in 32 fractions was delivered to the bilateral pelvic nodes. No concurrent chemotherapy was given.

The patient tolerated therapy without significant problems although he reported mild diarrhoea for which he did not require medication.

This patient’s follow-up CT scan unfortunately showed retroperitoneal lymphadenopathy. A CT PET scan was performed to see if this was solitary metastatic disease and therefore to assess his suitability for salvage therapy. Unfortunately, the CT PET scan, performed 5 months after treatment, showed extensive retrocrural and supraclavicular lymphadenopathy. Eight months after completing radical treatment he continues on palliative carboplatin and gemcitabine therapy.

Table 1 provides a summary of patient characteristics, treatment parameters and outcomes for ease of comparison.

**Table 1.** Summary of patient characteristics, treatment parameters and outcomes

Patient number	1	2	3	4	5
Age at diagnosis (year)	79	77	74	67	70
Performance status	1	1	1	1	0
Sex	Male	Male	Male	Female	Male
Neoadjuvant chemotherapy	Yes	Yes	Yes	Yes	Yes
Concurrent chemotherapy	Yes	Yes	Yes	Yes	No
Radiotherapy dose and fractionation	64 Gy/32# to bladder 53 Gy 32# to nodes	64 Gy/32# to bladder and nodes	64 Gy/32# to bladder 53 Gy 32# to nodes	Bladder 64 Gy/32# Suspicious pelvic node 52 Gy/27# Bilateral nodal volume 48 Gy/ 27#	64 Gy/32# to bladder 53 Gy 32# to nodes
Platform	Tomo	V-MAT	Tomo	V-MAT	Tomo
Small bowel dose reported	NA	N	V40 = 77.6%	V45 = 43.6 mm^3^	V40 = 19.2%
Rectal dose reported	V50 = 13.7%	NA	V50 = 28.8%	V50 = 33.1%	V50 = 7.2%
Femoral head dose reported	Mean left = 47.7% Mean right = 47.8%	NA	Left and right V50 = 0.0%	Left V50 = 0.0% Right V50 = 12.4%	Left and right V50 = 0.0%
Toxicity reported	Mild diarrhoea; ThromP	ThromP	ThromP	Nil	Mild diarrhoea
Outcome at last follow-up	No concerns	No concerns	No concerns	Metastatic disease	Metastatic disease

#, fractions; NA, not applicable; Tomo, tomotherapy; ThromP, thrombopenia.

### Radiotherapy planning

All patients were treated with IMRT. Contouring of gross tumour volume, clinical target volume (CTV) and PTV was carried out using Prosoma software and planning was performed on Monaco® (Elekta, Stockholm, Sweden) or Accuray Tomotherapy® (Sunnyvale, CA, USA) programme as appropriate. Three patients were treated on a Tomotherapy platform and two using volumetric-modulated arc therapy (V-MAT – Elekta). The doses to the PTV were excellent. On average 99% of the whole bladder received a dose of 61.36 Gy (range 61.0–61.6 Gy). The pelvic nodes were also treated well with doses up to an average of 53.74 Gy to 99% of the volume (range 50.8–61.7 Gy). Positive nodes were treated to up to 51 Gy.

No standardized treatment protocol is in place for this treatment; however, organs at-risk volume included rectum, bowel and femoral heads. The rectal dose varied with V50 (volume receiving 50 Gy) between 7.2% and 38.5%. The average was 26.1%. Bowel dose was not always calculated. However, the V40 average was 46.6% (range 19–77%). Only one plan required a concession value of the constraints for femoral head doses with a V50 of 12.4%. All other patients met the initial standard with a V50 of less than 5%.

**Figure 1.**
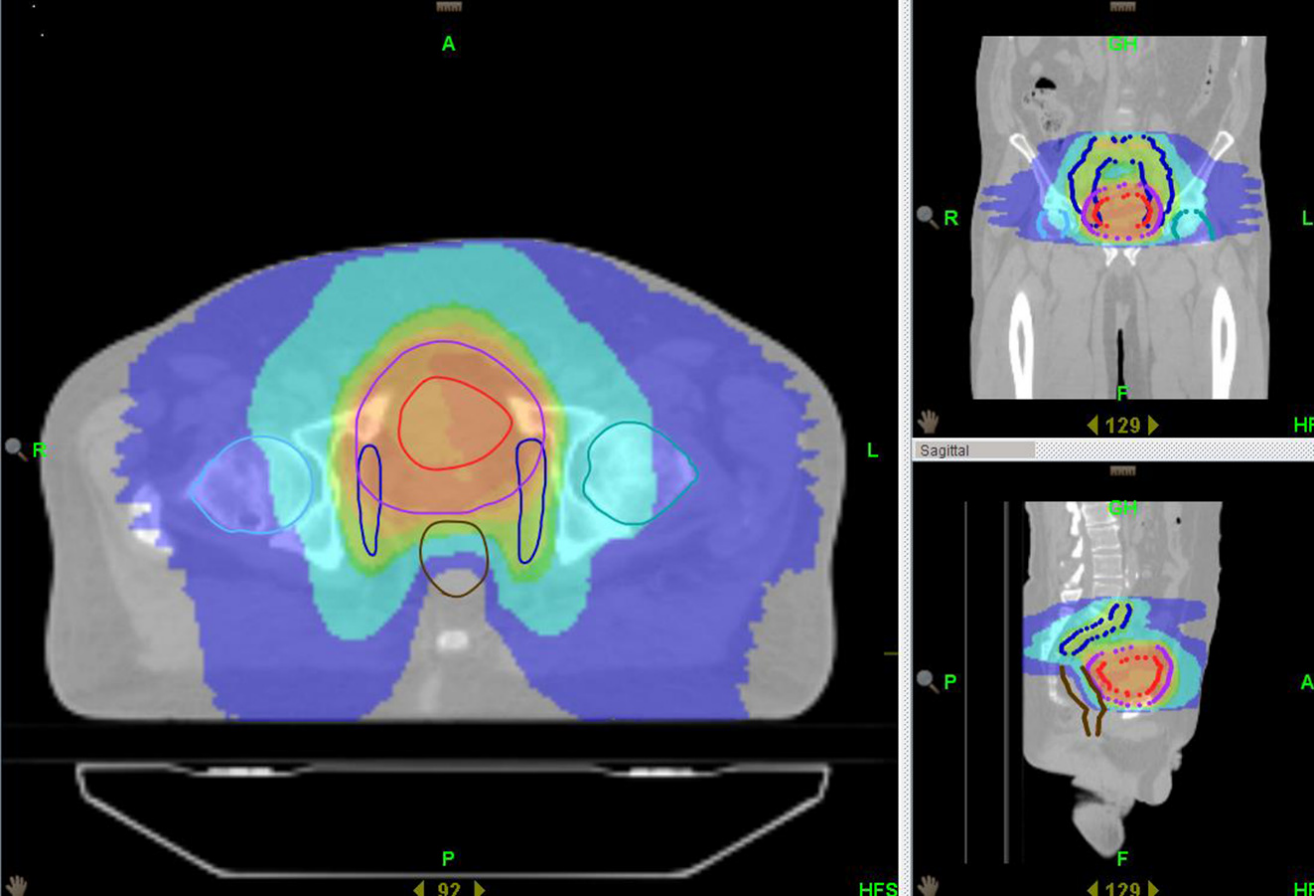
Tomotherapy plan.

**Figure 2.**
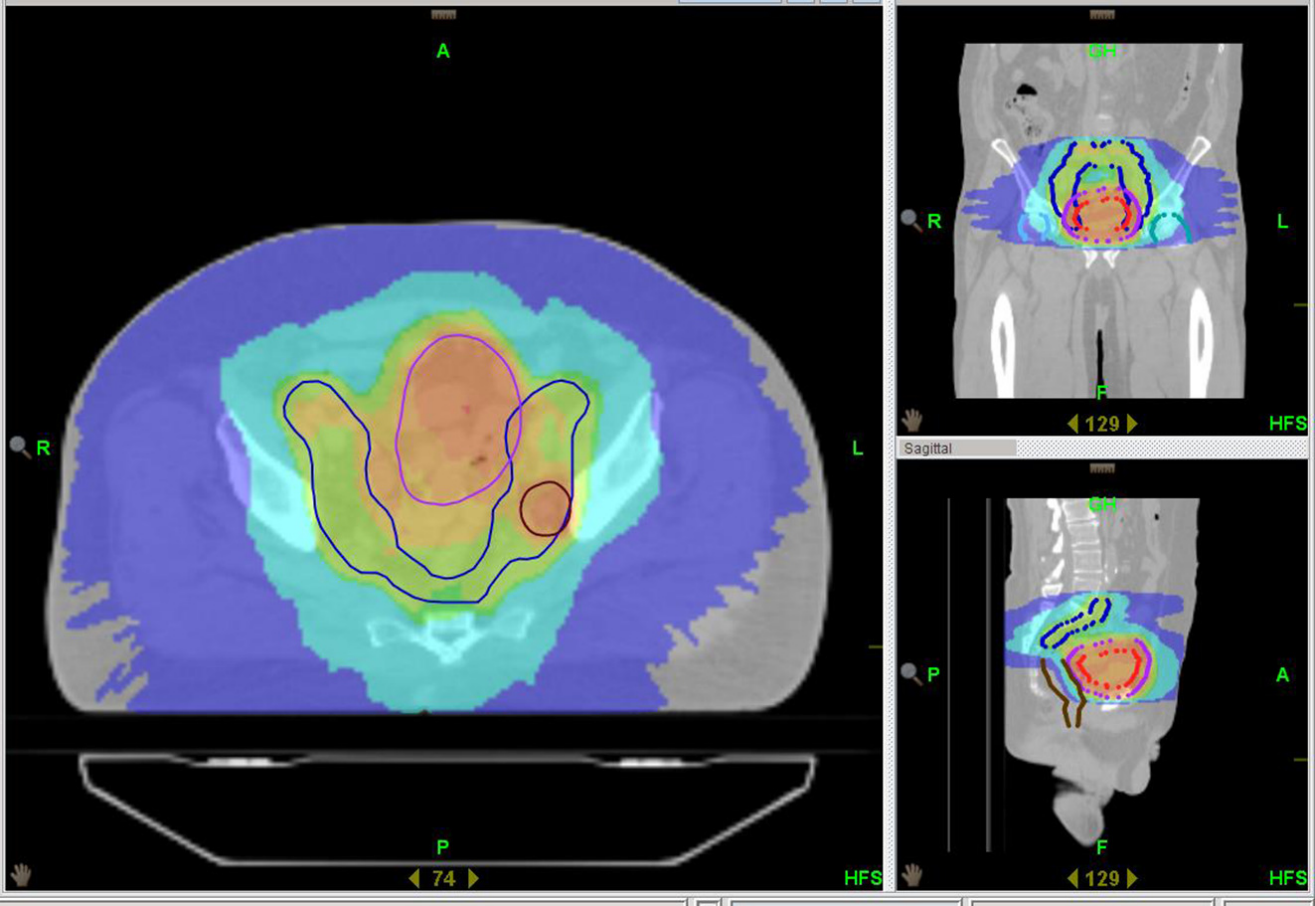
Tomotherapy plan.

Figures 1 and 2 show dose distributions from a patient treated on Tomotherpay. Dark blue is 19 Gy; sky blue is 32 Gy; green is 50 Gy, orange is 57 Gy and red is 64 Gy. The contoured volumes visible are the bladder PTV in purple and pelvic nodes in deep blue (with an involved node in brown in the upper pelvis axial slice). On the lower pelvic slice the bladder CTV is in red and PTV in purple. Pelvic nodes are in blue. The rectum and femoral head volumes are also shown.

**Figure 3.**
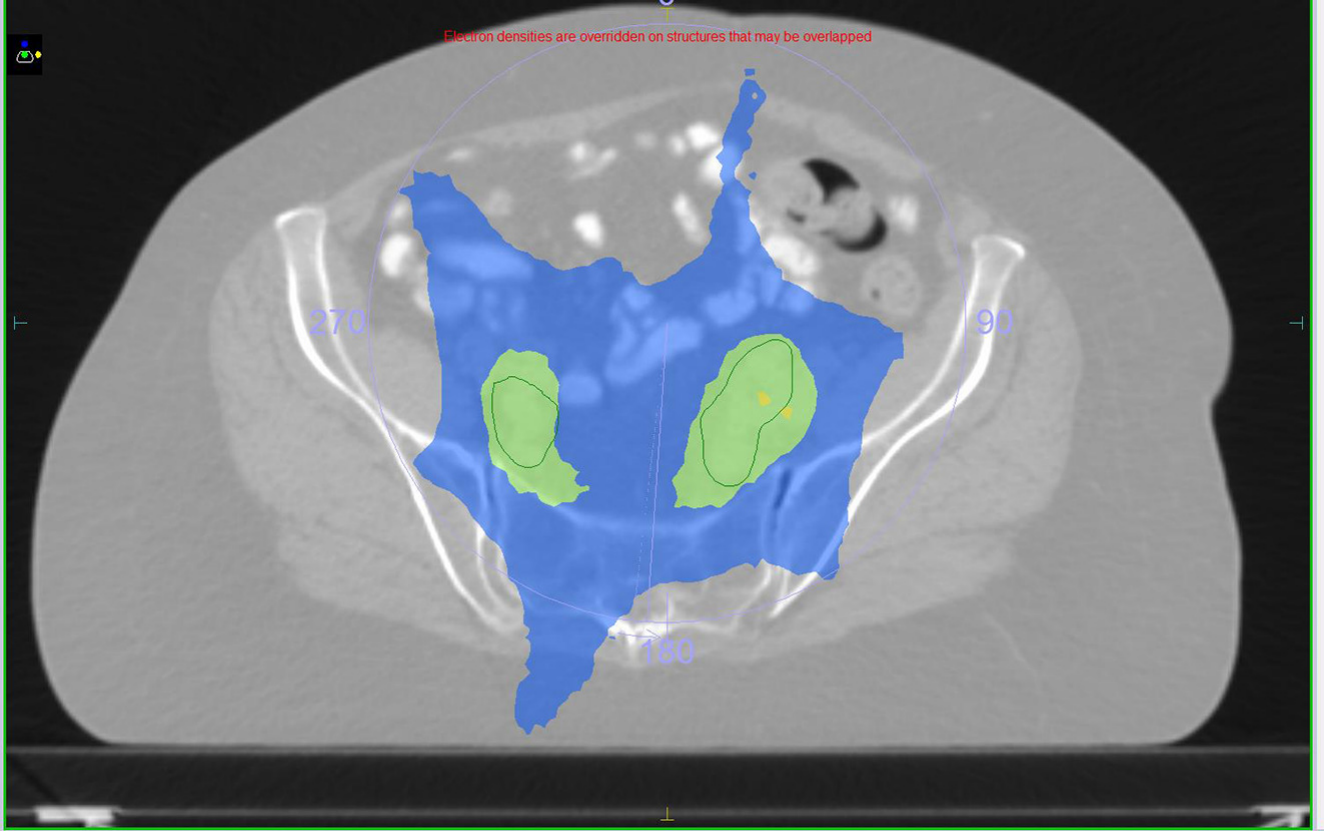
V-MAT plan.

**Figure 4.**
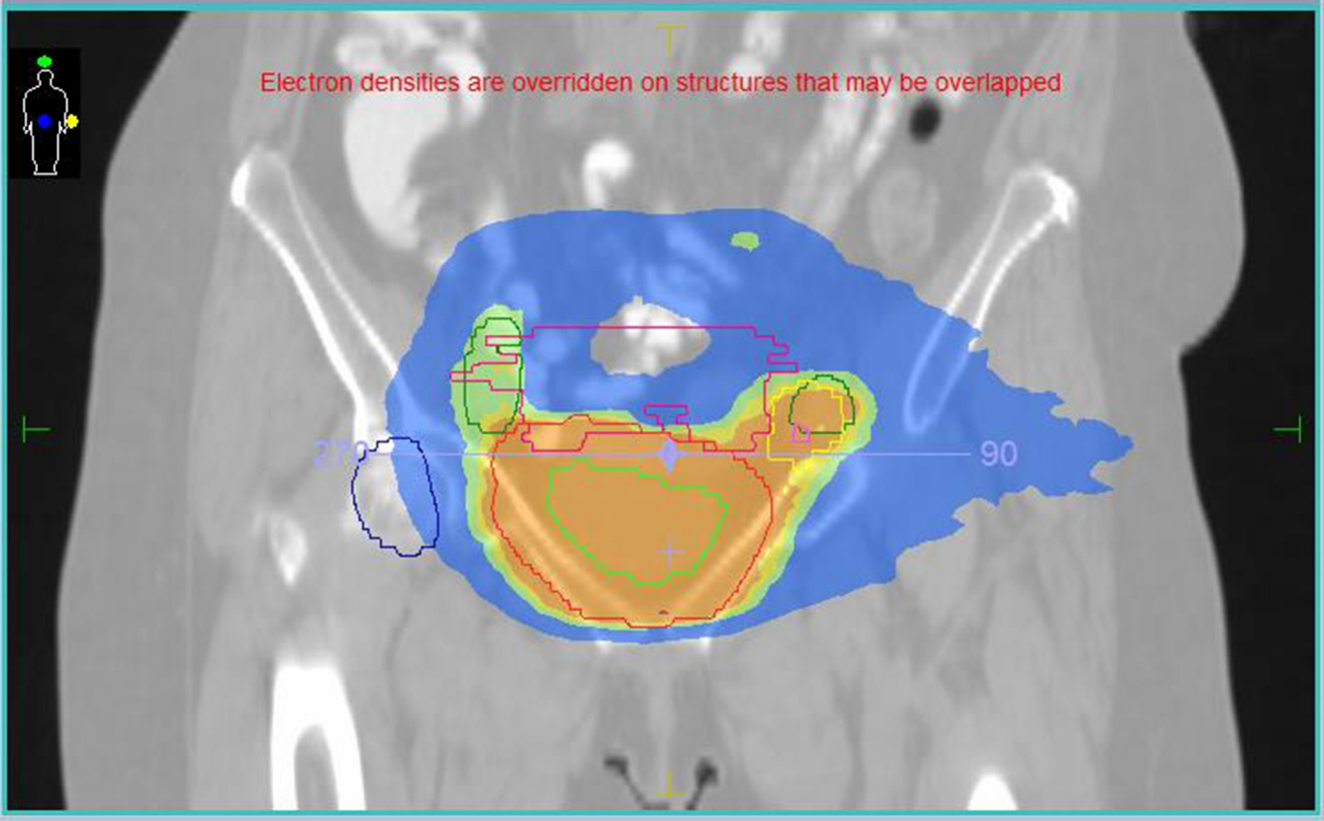
V-MAT plan.

**Figure 5.**
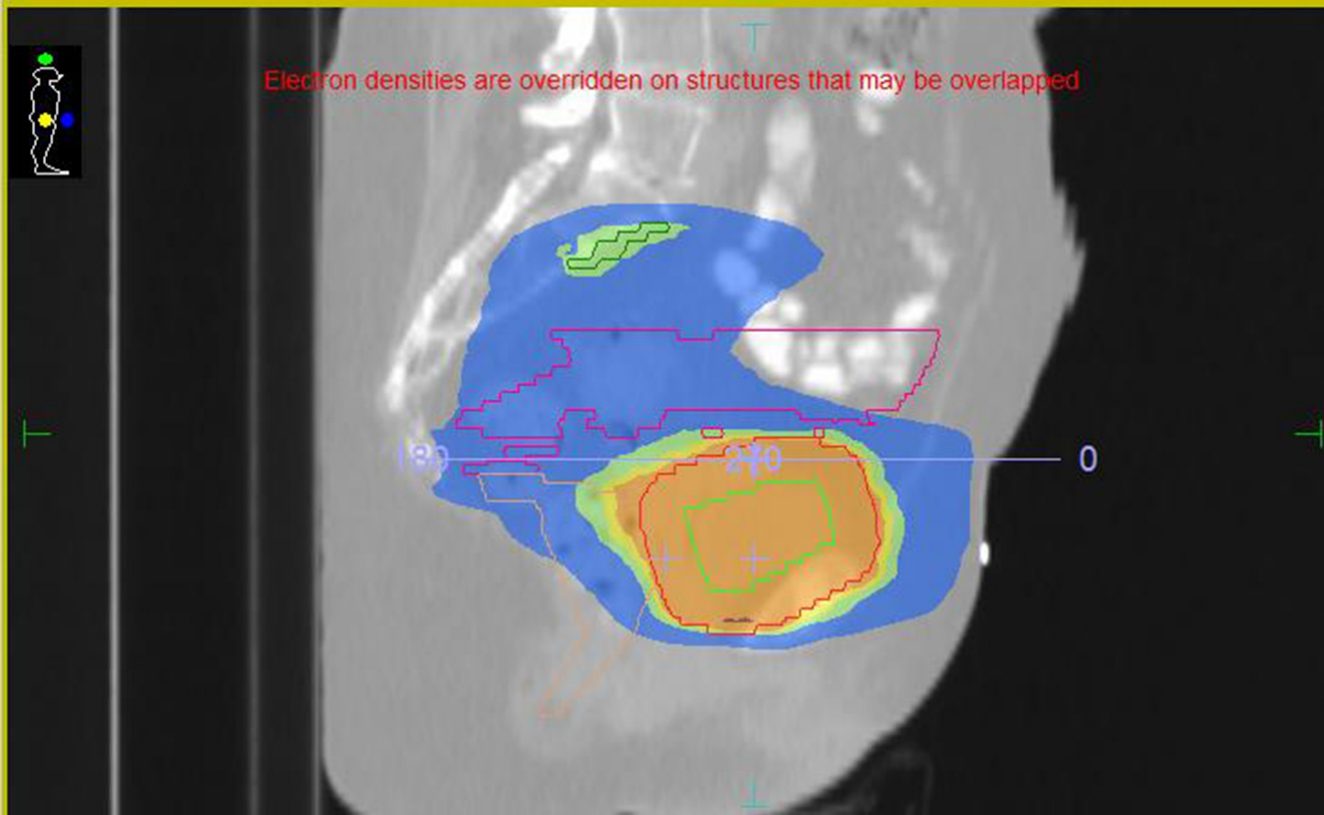
V-MAT plan.

Figures 3–5 show one of the V-MAT plans. Sky blue indicates a dose of 32 Gy and green 45 Gy. In the coronal and sagittal slices higher doses of 49 Gy (yellow) and 51 Gy (orange) are shown. Bladder CTV is contoured in red, nodal volume is seen in green and bowel shown in pink.

Image-guided radiotherapy was used for all five patients. Those treated on Tomotherpay were imaged using megavoltage CT. Those treated on V-Mat were imaged with kilovoltage cone beam CT. All patients were imaged before each treatment. This increases both time of delivery and radiation received but was felt necessary given the potential variation in bladder volume between fractions.

## Conclusions

All patients were made aware that treatment was not standard including the possibility that a larger radiation volume may increase toxicity. Appropriate consent was taken from all of them. On the whole, treatment was tolerated well with only minimal gastrointestinal and urinary symptoms reported. Three of the five patients had thrombocytopenia of note; all three of these patients were receiving concomitant chemotherapy. Only one of the four patients who received chemoradiotherapy did not experience a fall in the platelet count. Thrombocytopenia was not reported in the BC2001 trial and the relative high incidence of this complication with our small patient group may be explained by the larger volume of radiation exposure. Such an observation may be due to patients receiving both neoadjuvant and concurrent chemotherapy, however. Further work could be aimed at reviewing the dose received by the pelvic bone marrow. This could investigate if the dose is high and given the concurrent chemotherapy could this explain the high incidence of thrombocytopenia?

Average follow-up is 11.8 months. Local control appears to be good with all of the five patients having no pelvic relapse at the time of reporting. Two patients have relapsed with distant metastatic disease. No long-term side effects of therapy have been reported. However, a relatively short median follow-up is not long enough to truly assess late presentations of radiotherapy toxicity.

All patients were staged thoroughly radiologically; however, it should be noted that nodal biopsies were not performed. It is possible that the observed lymphadenopathy was reactive. It is not routine to biopsy pelvic lymph nodes unless there is doubt as to whether they are involved. The multidisciplinary teams involved in each case were satisfied that the enlarged nodes identified were due to metastatic disease.

The patients treated were selected for this aggressive management based on their excellent performance status (WHO 0 or 1 for all five patients) and their otherwise unremarkable past medical history. Such extensive radiotherapy is not suitable for all patients but, in a select few, may prove to be a suitable and successful treatment option.

IMRT techniques allow larger volumes to be treated owing to improved conformality and the resulting reduced toxicity. As seen in other cancer sites, such as prostate, anal and rectal malignancy, radiation to pelvic nodes appears appropriate in a carefully selected group of patients. Treatment may be appropriate with both radical and adjuvant doses of radiation. For example, patients with node-positive disease could be treated with an integrated boost to positive nodes and high-risk patients could receive an adjuvant dose to pelvic nodes. More work is necessary to assess which patients would benefit and are most suitable for such treatment. A treatment protocol should be developed to ensure consistent reporting of doses prescribed and achieved for both the target volumes and organs at risk. Future trials should be powered sufficiently to assess efficacy and also provide adequate follow-up to evaluate long-term bowel toxicity.

## Learning points

1. Radiotherapy to the bladder and pelvic nodes is well tolerated and can be used as a treatment option, with or without chemotherapy, for patients with node-positive bladder cancer.
2. Treating with IMRT allows for an appropriate dose distribution that keeps radiation dose within the tolerance of other pelvic structures.
3. Patients may experience greater thrombocytopenia during this treatment – possibly owing to the larger volume of bone marrow exposed to radiation.

## Consent

Written informed consent for the case to be published (including images, case history and data) was obtained from the patient(s) for publication of this case report, including accompanying images.
